# Development of a prototypic, field-usable diagnostic tool for the detection of gram-positive cocci-induced mastitis in cattle

**DOI:** 10.1186/s12917-024-04028-5

**Published:** 2024-05-02

**Authors:** Anna Dobrut, Jakub Skibiński, Adrian Bekier, Kamil Drożdż, Karolina Rudnicka, Przemysław Płociński, Izabela Siemińska, Monika Brzychczy-Włoch

**Affiliations:** 1 Department of Molecular Medical Microbiology, Chair of Microbiology, Jagiellonian University Medical College, Krakow, Poland; 2 BioMedChem Doctoral School of University of Lodz and Lodz Institutes of The Polish Academy of Sciences, University of Lodz, Lodz, Poland; 3 Department of Immunology and Infectious Biology, Faculty of Biology and Environmental Protection, University of Lodz, Lodz, Poland; 4 Department of Immunology and Allergy, Chair of Pulmonology, Rheumatology and Clinical Immunology, Medical University of Lodz, Lodz, Poland; 5 Institute of Veterinary Sciences, University Center of Veterinary Medicine JU-AU, University of Agriculture in Krakow, Krakow, Poland

**Keywords:** Bovine mastitis, Lateral flow immunoassay, *Streptococcus uberis*, *Streptococcus agalactiae*, *Staphylococcus aureus*, Elongation factor Tu

## Abstract

**Background:**

Bovine mastitis is one of the most widespread diseases affecting cattle, leading to significant losses for the dairy industry. Currently, the so-called gold standard in mastitis diagnosis involves determining the somatic cell count (SCC). Apart from a number of advantages, this method has one serious flaw: It does not identify the etiological factor causing a particular infection, making it impossible to introduce targeted antimicrobial therapy. This can contribute to multidrug-resistance in bacterial species. The diagnostic market lacks a test that has the advantages of SCC and also recognizes the species of pathogen causing the inflammation. Therefore, the aim of our study was to develop a lateral flow immunoassay (LFIA) based on elongation factor Tu for identifying most prevalent Gram-positive cocci responsible for causing mastitis including *Streptococcus uberis, Streptococcus agalactiae* and *Staphylococcus aureus*.

**Results:**

As a result, we showed that the assay for *S. uberis* detection demonstrated a specificity of 89.02%, a sensitivity of 43.59%, and an accuracy of 80.3%. In turn, the second variant - assay for Gram-positive cocci reached a specificity of 95.59%, a sensitivity of 43.28%, and an accuracy of 78.33%.

**Conclusions:**

Our study shows that EF-Tu is a promising target for LFIA and we have delivered evidence that further evaluation could improve test parameters and fill the gap in the mastitis diagnostics market.

**Supplementary Information:**

The online version contains supplementary material available at 10.1186/s12917-024-04028-5.

## Background

Bovine mastitis is one of the most common diseases worldwide, affecting dairy cattle and generating enormous financial losses for the dairy industry – even 2 billion dollars annually in the USA, which is 385 to 770 dollars per cow [1, 2]. In turn, according to estimates, the losses due to mastitis in European cows may amount to as much as 240 euros per cow per year [3]. Mastitis can take the form of a clinical, sub-clinical, or chronic infection, depending on the pathogenicity of the bacteria causing the infection and the animal’s age, lactation status, and immunological status. The clinical form of mastitis (CM) is considered the most severe and symptomatic, as it presents visible and palpable symptoms such as pain, swelling, redness of the udder, elevated temperature or fever, a change in the appearance of milk, and a reduction in milk production [4, 5]. The frequency of CM varies regionally, ranging from 12 to 30% [6]. Due to the lack of visible symptoms, the sub-clinical form of mastitis is most challenging to diagnose and treat. Undiagnosed chronic asymptomatic mastitis generates economic loss related to the decrease of milk production with an increase in the somatic cell count. The loss contributed by sub-clinical mastitis accounts for more financial losses in the herd than do clinical cases. The chronic form is the least common, but may lead to persistent infection as well as long-term stimulation of the immune system [7]. Regardless of the form, bacteria are the most common etiological agents of mastitis, with fungi and algae being rarely detected. Among most commonly detected bacteria causing mastitis are *Streptococcus uberis*, *Staphylococcus aureus*, *Streptococcus dysgalactiae*, *Streptococcus agalactiae*, non-aureus Staphylococci (NAS), *Corynebacterium bovis*, *Mycoplasma bovis*, and Gram-negative rods, such as *Escherichia coli* and *Klebsiella pneumoniae* [8, 9].

The simplest and cheapest form of mastitis diagnosis that uses somatic cell count (SCC) is the California Milk Test. This assay can be carried out even by the milker and the result is available immediately. If the milk gels, this means a significant presence of SCC and identifies exactly which quarter the problem lies in [10]. This widely used method demonstrates a number of advantages. Undoubtedly, the most significant advantage of this approach is its capability to rapidly identify mastitis in livestock directly in the field. This enables the infected cow to be immediately separated from the herd and empirical antimicrobial therapy to be introduced. As a result, it also leads to lower financial losses [11]. Nevertheless, the key limitation of this method is the inability to identify the etiological factor. Without knowing which specific pathogen is causing an infection, one cannot choose a targeted antibiotic therapy. Administering an antimicrobial therapy without identifying the specific pathogen may result in selection of the resistant bacteria and may increase the number of multidrug-resistant bacterial strains, which is a growing problem worldwide [12].

Common methods for identifying the microbial etiological agent – including bacteriological identification (isolation and biochemical identification), molecular biology-based assays, and serological or spectrometric methods (including MALDI-ToF) – demonstrate a number of disadvantages. These diagnostic methods are more time-consuming and require specialized equipment and qualified personnel to perform the tests and interpret the results. In addition, the results are often difficult to interpret unambiguously. However, most importantly, they cannot be performed outside the laboratory, which significantly limits their usefulness in the rapid diagnosis of cattle diseases [13–15].

To solve the issues connected with current identification methods and to combine the advantages of SCC tests, scientists have been working on the development of an immunochromatographic assay (lateral flow immunoassay [LFIA]), which addresses the lack of a rapid diagnostic tool for microbe-derived mastitis. This diagnostic test facilitates the expeditious (within a few minutes) identification of pathogens in various biological specimens (such as milk, blood, and stool) and – more importantly – does not require specialized equipment, is easy to perform, and is simple to interpret. However, the biggest advantage is that lateral flow immunoassay can be performed directly in the field, not only by the veterinarian, but also by cattle breeders. In combination with the low cost, in comparison with other molecular and serological methods, this technique proves to be more accessible [16]. The greatest difficulty in developing an effective LFIA test is selecting appropriate targets (antigens) recognized by defined antibodies, which directly impacts the sensitivity and specificity of the test. For this purpose, bioinformatic prediction should be carried out and followed by in vitro tests [17, 18].

An antigen that might be considered a potential target for immunochromatographic assay is elongation factor thermo unstable Tu (EF-Tu), one of the most abundant and conserved bacterial proteins. This moonlight protein, approximately 44 kDa, is involved in several pathogenic functions, such as invasion, adhesion, and modulation of the humoral immune responses [19–24]. EF-Tu is a common protein for eucaryotic and procaryotic organisms, constituting up to 5% of the total cell content [20, 25]. In the bacterial cell, this protein constitutes part of the membrane cytoskeleton and is involved in the elongation phase of de novo protein synthesis and in the translation process in prokaryotic cells. Even though EF-Tu is a component of the membrane cytoskeleton, it requires localization on the cell surface. EF-Tu does not contain a signal secretion motif, which explains the moonlight character of this protein [26, 27]. Several paths of EF-Tu translocation outside the bacterial cell have been described viz., extracellular vesicle secretion, cell lysis, and the Sec machinery 54, which involves other proteins [28–30]. EF-Tu is also known for its immunogenic role [31–36], as demonstrated in our previous studies [17, 37]. The main concern about EF-Tu is its conservative nature, which can result in cross-reactivity between different bacterial species; nevertheless, our previous studies carried out on serum samples from cows with mastitis caused by several bacterial species have delivered promising results. We did not observe cross-reactivity between proteins obtained from bacterial isolates isolated from the milk samples with confirmed mastitis caused by *Streptococcus* (*S. uberis*, *S. agalactiae*, *S. dysgalactiae*) [17]. Therefore, the current study is intended to examine the usefulness of this protein as an antigen for LFIA prototype in the diagnosis of bovine mastitis caused by Gram-positive cocci, including *S. uberis*, the most common bacterial species responsible for mastitis in Polish cattle. We also attempted to verify the utility of this target for multiplex assay in screening for mastitis caused by *S. uberis* and/or *S. agalactiae* and/or *S. aureus*. This approach can be advantageous, as several bacteria species, including *S. uberis, S. agalactiae*, and *S. aureus* can cause mastitis. The treatment of mastitis infection is based on antibiotic administration. However, the extensive use of antibiotics induces resistance in pathogens and lowers the efficiency of eradication. Therefore, the precise identification of causative microorganisms in one multiplex assay can lead to the use of more specific antibiotics, which raise the successful rate of eradication and prevent the formation of resistant bacterial strains.

## Results

### Determination of optimal condition of anti-EF-Tu monoclonal antibody

**Fig. 1:**
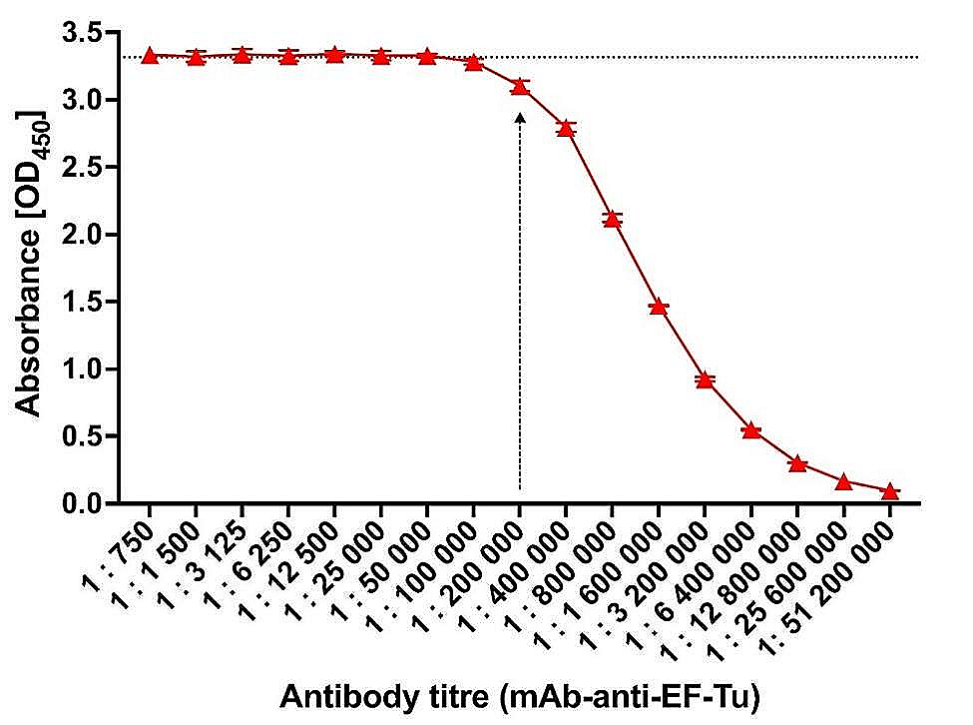
ELISA titer graph of mAb-anti-EF-Tu generated against recombinant EF-Tu. Each point represents the combined averages of three independent experiments in triplicate under the same conditions. Error bars indicate standard deviation

Based on the indirect ELISA, we first determined the monoclonal antibody titer. At different dilutions (1:50–1:51,200,000), the antibodies reacted with an equal amount of the recombinant EF-Tu protein. The antibody titer was defined as the dilution of the antibody corresponding to the decrease in absorbance at 490 nm. The curve indicates the change in absorbance at a value that corresponds to antibody dilution times of about 200,000, as graphically summarized in Fig. 1.

**Fig. 2:**
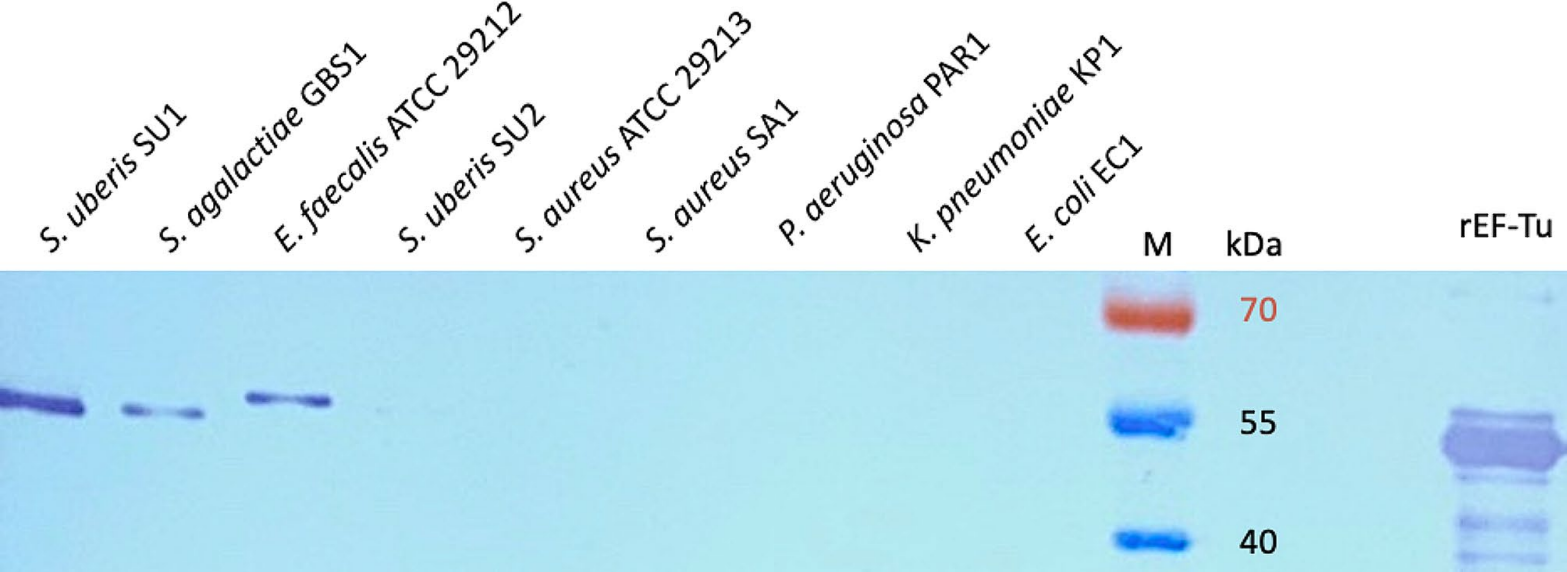
Western blot analysis of bacterial lysates, the source of native EF-Tu, and recombinant EF-Tu (rEF-Tu) protein, both recognized by monoclonal antibodies against elongation factor-Tu

Western blot analysis was performed to confirm the concept that mAb-anti-EF-Tu generated against recombinant EF-Tu also binds to the native elongation factor-Tu protein, but only in *Streptococcus* species. For this purpose, we used lysates from various bacterial strains: *S. uberis* SU1, *S. agalactiae* GBS1, *Enterococcus faecalis* ATCC 29,212, *S. uberis* SU2, *S. aureus* ATCC 29,213, *S. aureus* SA1, *P. aeruginosa* PAR1, *K. pneumoniae* KP1, and *E. coli* EC1. The Western blot analysis revealed the recognition of both recombinant and native EF-Tu by mAb-anti-EF-Tu, resulting in bands with a molecular mass of approximately 60 kDa. Strong bands were visible on the following test lines: *S.uberis* SU1, *S. agalactiae* GBS1, and *E. faecalis* ATCC 29,212, but a slight smear also appeared on the *S. uberis* SU2 test line (Fig. 2).

**Fig. 3:**
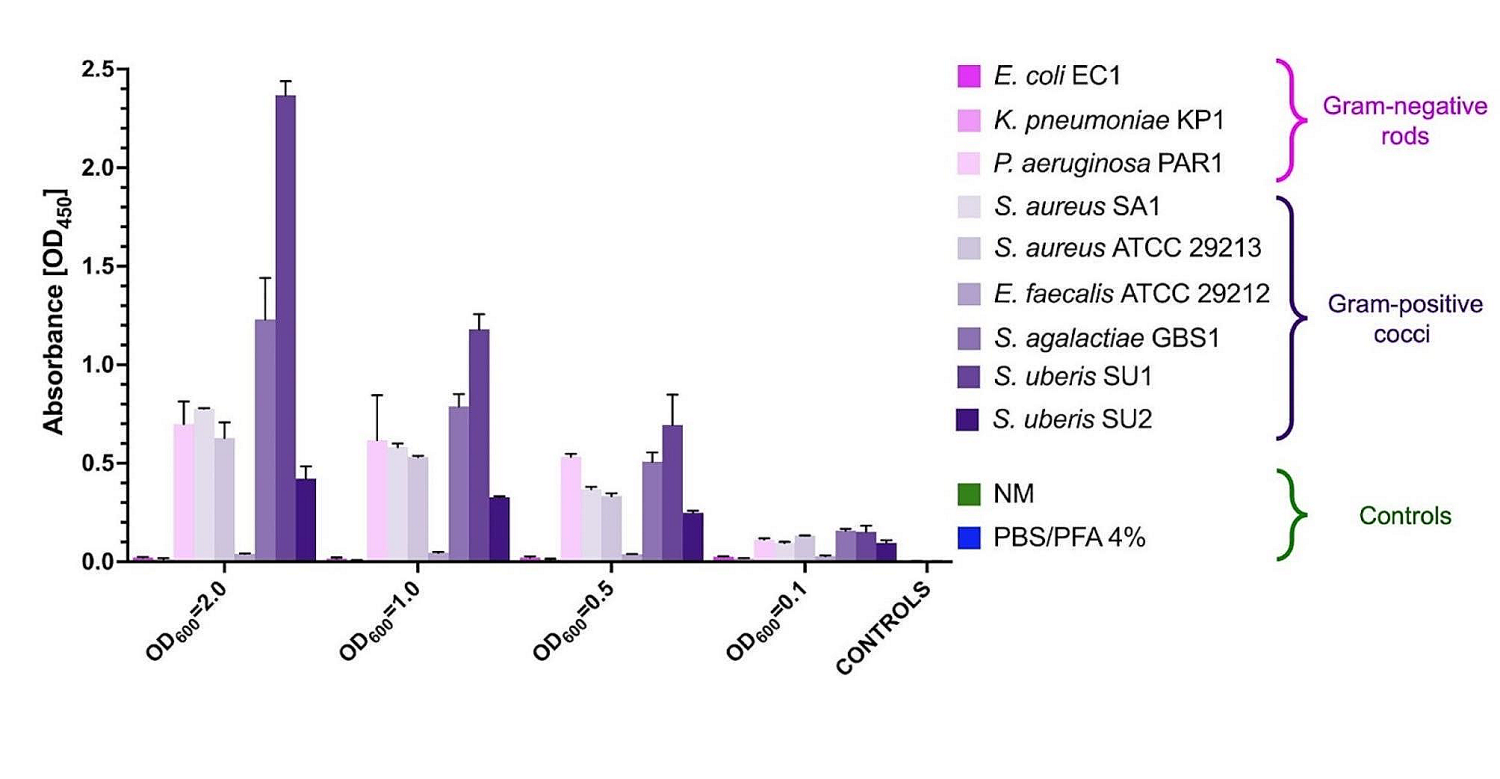
cELISA graph of mAb-anti-EF-Tu generated against whole bacteria and milk from cows without mastitis. Each value represents the combined averages of three independent experiments in triplicate under the same conditions. Error bars indicate standard deviation. Legend: NM – negative milk, PBS/PFA 4% – fixative, negative control

In the final step, we performed cellular ELISA (cELISA) to investigate the binding capacity of mAb-anti-EF-Tu to bacteria. For this purpose, we used whole bacteria, the same strains as in the Western blotting. As a negative control, we used milk obtained from cows without diagnosed mastitis. The mAb-anti-EF-Tu bound to Gram-positive cocci, which also confirmed the results from Western blotting. Moreover, cELISA showed that the mAb-anti-EF-Tu for detecting *S. uberis* bound to both reference *S. uberis* strains. Also, the results indicated that non-specific reactions between monoclonal antibodies (mAb-anti-EF-Tu) and components of milk from cows without mastitis were excluded **(**Fig. 3**)**.

To summarize, the Western blot band corresponding with native EF-Tu protein appeared for the reference strains of *S. uberis* SU1, *S. agalactiae* GBS1, *E. faecalis* ATCC 29,212, and *S. uberis* SU2, which are Gram-positive cocci. Moreover, cELISA showed that the anti-EF-Tu mAb for detecting *S. uberis* strongly bound to the reference *S. uberis* SU1 strains, which were selected to standardize the procedure of LFIA. The nonspecific binding *to P. aeruginosa* received special attention in the prototype version of the LFIA.

### Characterization of gold nanoparticles (AuNPs)

**Fig. 4:**
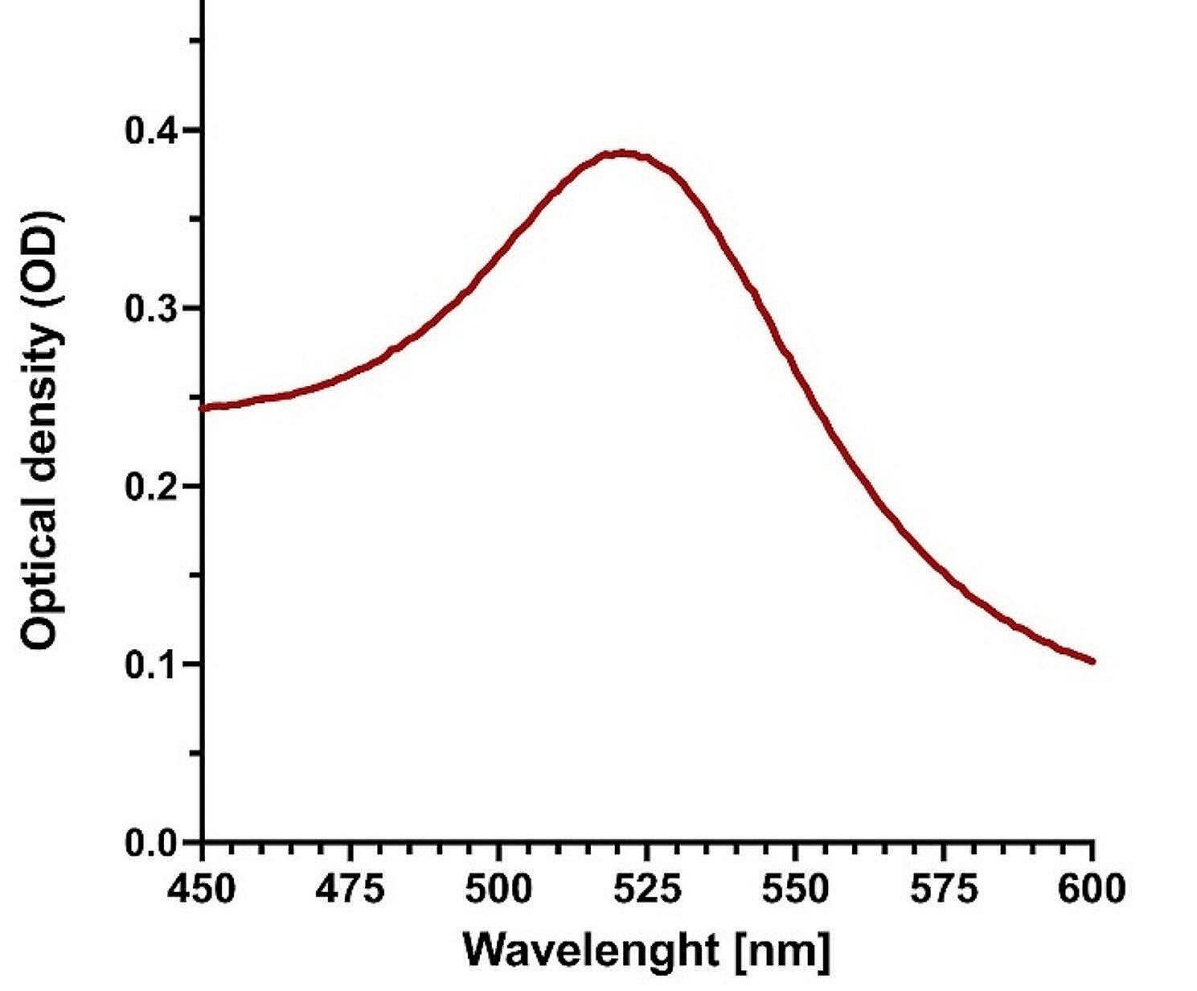
Particle size analyzer graph for UV-Vis spectra of 30 nm AuNPs

The colloidal AuNPs as a label for LFIA were successfully synthesized by chemical reaction involving reduction of tetrachloroauric (III) acid with sodium citrate. The seed size of 30 nm AuNPs was confirmed by UV-Vis spectroscopy analysis. The maximum peak at absorbance (OD) around 0.4 was at wavelength (λmax) 526 nm, which confirmed the size of our AuNPs (Fig. 4).

### Assessment of conjugation conditions and conjugate stability

**Fig. 5:**
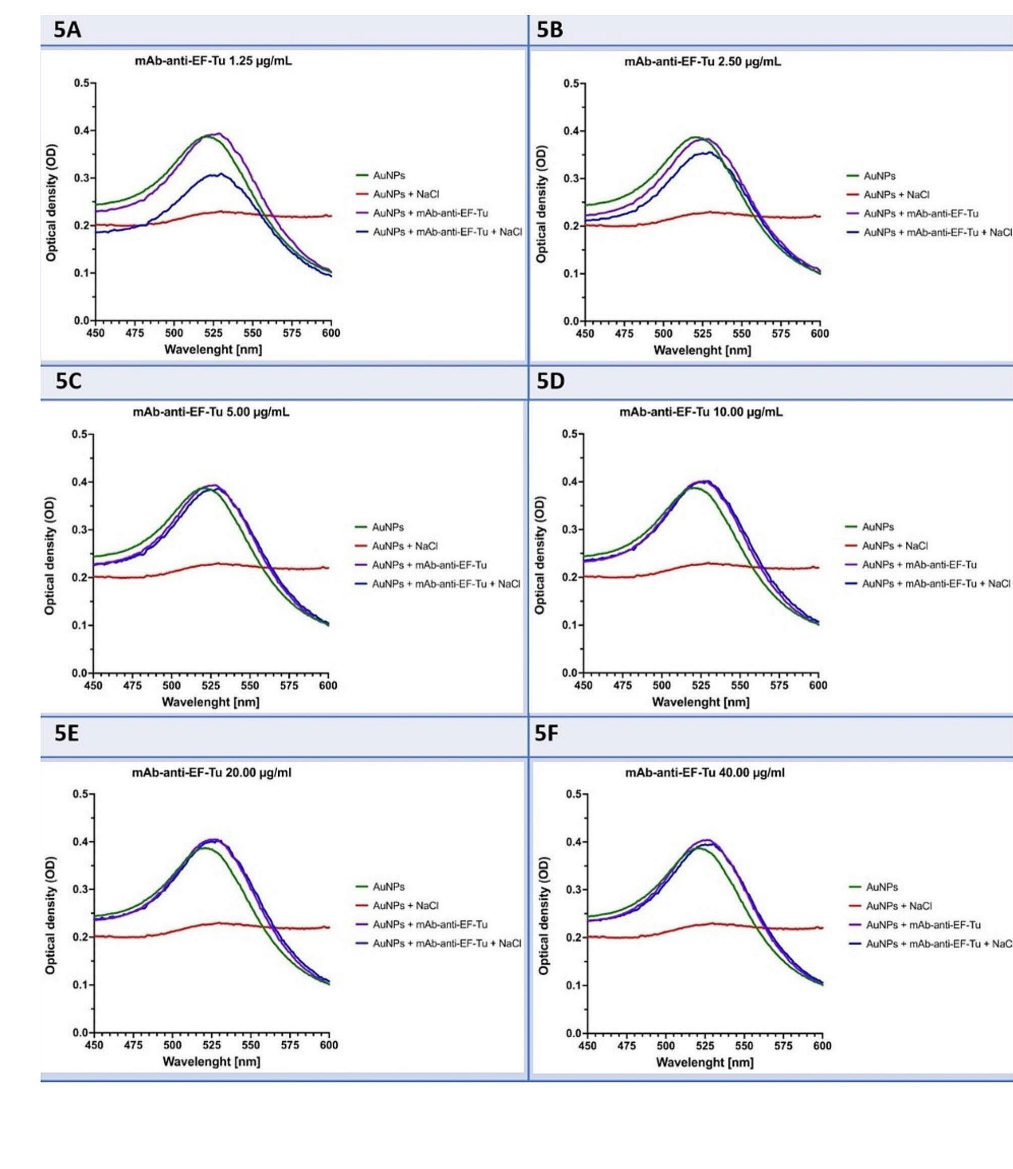
The stability of AuNP–antibody conjugates for UV-Vis spectra at different concentrations of mAb-anti-EF-Tu (1.25 µg/mL **(5A)**, 2.5 µg/mL **(5B)**, 5 µg/mL **(5C)**, 10 µg/mL **(5D)**, 20 µg/mL **(5E)**, and 40 µg/mL **(5F)**. Each point represents the combined averages of three independent experiments in triplicate under the same conditions

The stability of the AuNP–antibody conjugates was visually assessed by measurements of changes in UV-Vis – absorbance (OD) following the addition of 1 M NaCl to the conjugate samples. AuNPs that had no salt or antibody were used as a reference for unstable conjugates. In cases where AuNP–antibody conjugates indicated instability, a decrease of the OD value was observed, while the stable conjugates after the addition of NaCl remained at the same OD value (Fig. 5). The spectrum peak shift to the right observed between AuNPs and AuNP–antibody conjugates also confirmed the successful conjunction of gold nanoparticles with antibodies by increasing their diameter. Based on the resulting graphs, the selected concentration of mAb-anti-EF-Tu for conjugation with AuNPs was 10–20 µg/mL.

**Fig. 6:**
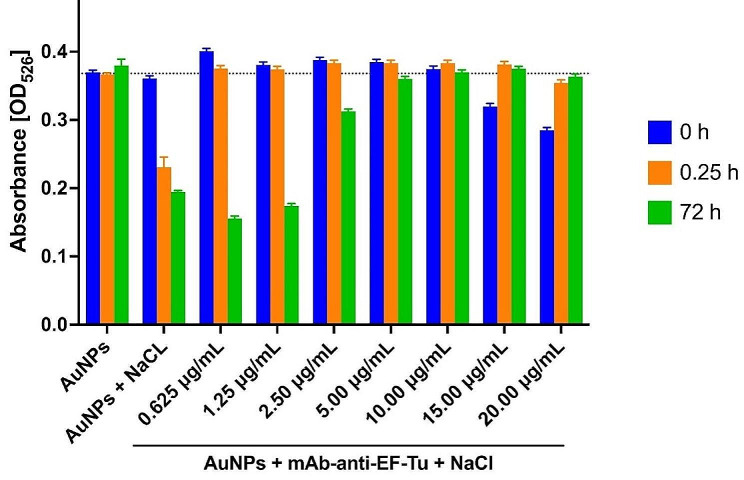
The stability of AuNP–antibody conjugates at different concentrations of mAb-anti-EF-Tu at three time-points. Each value represents the combined averages of three independent experiments in triplicate under the same conditions

Additionally, UV-VIS analysis determined the optimal concentration of mAb-anti-EF-Tu for conjugation with AuNPs, where the AuNP solution remained stable and no aggregates or precipitation of AuNPs occurred for up to 72 h. The optimal concentration of mAb-anti-EF-Tu used for conjugation with AuNPs was chosen based on the absorbance measurements (Fig. 6).

In the current study, the conjugates were mostly stable at a pH of 8.0 with the addition of 10 µg/mL of mAb-anti-EF-Tu; these conditions were selected for conjugation when producing the detection probes.

### Lateral flow immunoassay

The technical aspects of lateral flow immunoassay were based on experiments related to the reactivity of mAb-anti-EF-Tu and the AuNP–antibody conjugation conditions. The test uses two lines: a test line with mAb-anti-EF-Tu and a control line with goat mAb against mouse antibodies.

**Fig. 7:**
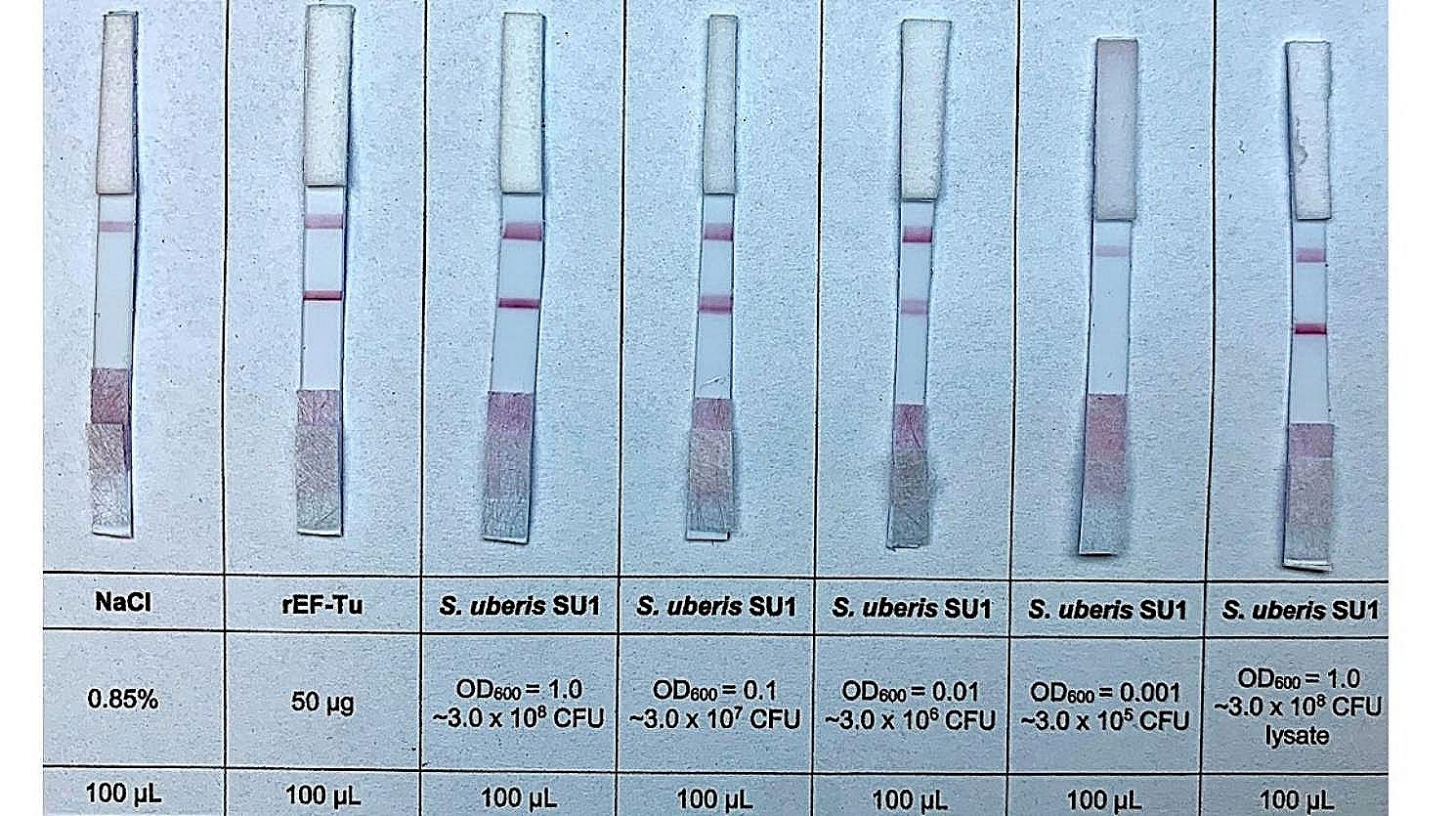
Sensitivity of LFIA strips to recombinant EF-Tu protein, different densities of live *S. uberis* SU1 and *S. uberis* SU1 lysate. rEF-Tu – recombinant EF-Tu protein

This lateral flow immunoassay was tested against recombinant EF-Tu protein (at a concentration of 50 µg per test), whole live *S. uberis* SU1 bacterial cells (OD_600_ = 1.0, OD_600_ = 0.1, OD_600_ = 0.01 and OD_600_ = 0.001) and the bacterial lysate of *S. uberis* SU1 (OD = 1.0). The results showed that the LFIA was designed and assembled correctly. The result was read after 15 min. The red control test line appeared in all negative and positive tests. Moreover, the strong red test bands were produced by samples with recombinant EF-Tu protein and *S. uberis* SU1 (OD 1.0). An identical result was obtained by the bacterial lysate of *S. uberis* SU1, the source of native EF-Tu protein. The visibility and intensity of the red test line were proportional to the amount of *S. uberis* SU1 in the sample. Thus, the minimal amount of *S. uberis* SU1 that produced a red test line detectable by the naked eye of three independent people (3.0 × 10^6^ CFU/test) was considered the visual limit of detection (Fig. 7).

### Characterization of milk samples

The characteristic by cultivation on ChromAgar mastitis of milk samples included in the investigation revealed that one hundred samples with clinical mastitis and 103 milk samples from cows that did not show symptoms of mastitis. Among the control group, 41 (42.7%) samples did not contain culturable bacteria, and 59 (57.3%) samples allowed the bacteria isolation. On the other hand, in the CM group, 25 (25.0%) milk samples did not contain culturable bacteria, and 75 (75.0%) yielded positive bacteria isolation. Bacteriological investigation of the clinical mastitis group revealed *S. uberis* as the predominant etiological agent, accounting for 38.0% (*n* = 38) of cases, followed by *E. coli* at 13% (*n* = 13), *S. agalactiae* at 10% (*n* = 10), and *S. aureus* at 8.0% (*n* = 8). Additionally, 15 samples (15%) exhibited other bacterial species including non-aureus *Staphylococci*, *Viridans Group Streptococcus*, and *Corynebacterium spp*. In the control group without clinical mastitis (*n* = 103), predominant species included NAS, *Viridans Group Streptococcus*, and *Corynebacterium spp.*, constituting 49.5% (*n* = 51) of samples, followed by *S. uberis* at 28.2% (*n* = 29), *S. aureus* at 9.7% (*n* = 10), and *E. coli* at 1.9% (*n* = 2). Notably, *S. agalactiae* was absent in the control group.

**Fig. 8:**
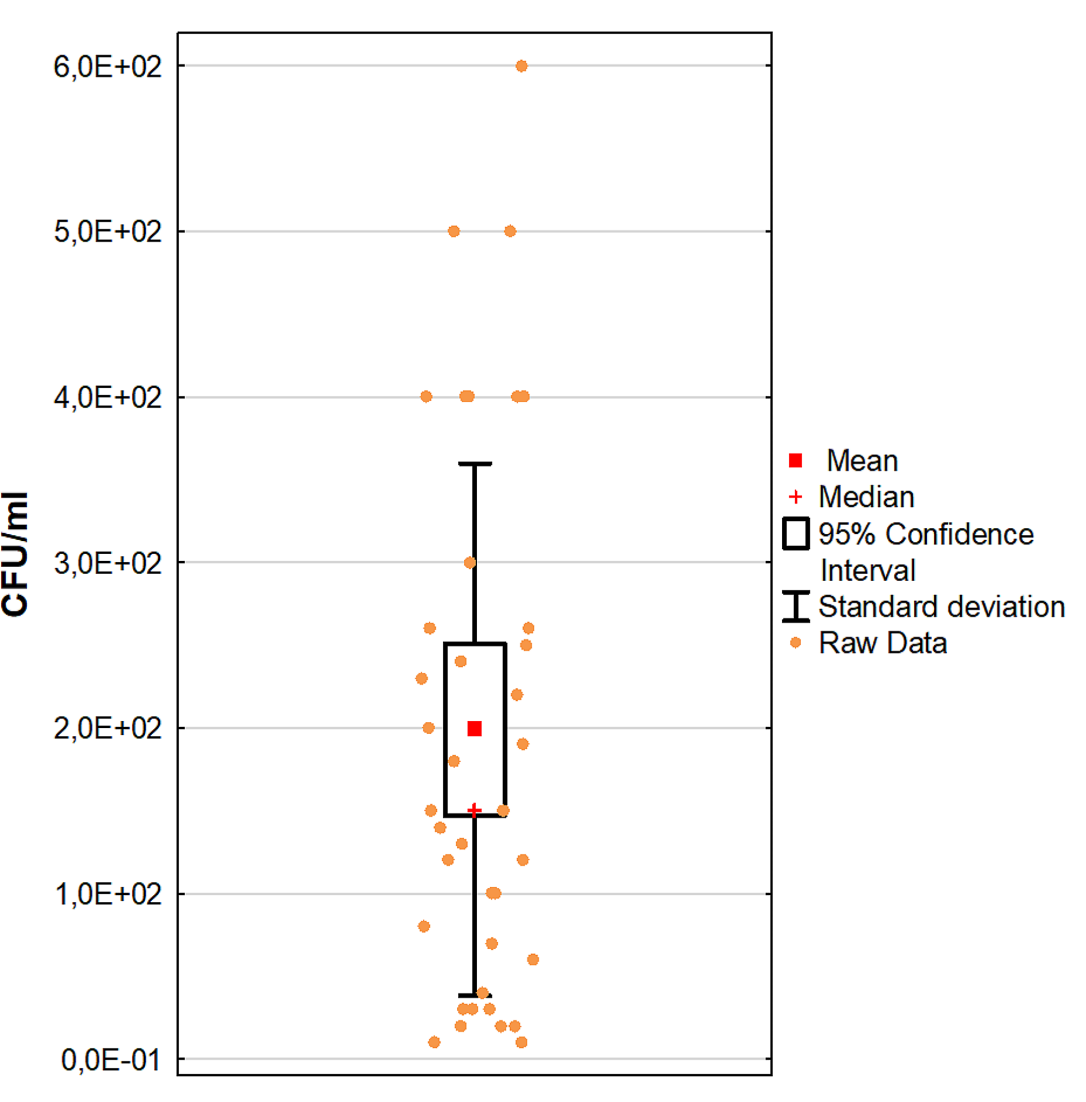
Mean values of bacterial numbers in milk samples infected with *S. uberis*. Legend: CFU – colony forming unit

Additionally, the colony forming unit (CFU/mL) was determined for each SU-positive sample. The mean CFU for all milk samples contaminated with *S. uberis* was 1.98 × 10^2^ CFU/mL (SD = 1.60 × 10^2^ CFU/mL, median 1.50 × 10^2^ CFU/mL) (Fig. 8). The results obtained by this method were treated as our gold standard and used to compare those obtained through LFIA.

### LFIA validation

The parameters of the LFIA based on recombinant EF-Tu protein and live *S. uberis* SU1 bacterial cells of known density defined in CFU/mL were validated by an examination of 203 milk samples. Next, the results were compared with our gold standard and subjected to statistical analysis. However, if no red line appeared at the control line, the result was considered invalid. The results are shown in Table 1. The analysis was performed in two approaches: (1) a monoplex diagnostic assay for the detection of *S. uberis* in bovine milk samples and (2) a multiplex assay for the detection of *S. uberis*, *S. agalactiae*, and *S. aureus* in bovine milk samples (Table 2). The second approach was considered due to the Western blotting and cELISA results. Those experiments showed that mAb-anti-EF-Tu were cross-reactive with some Gram-positive cocci, such as *S. aureus* SA1 and ATCC29213, *S. agalactiae* GBS, and *E. faecalis* ATCC 29,212. Also, to exclude the nonspecific binding of *P. aeruginosa* to mAb-anti-EF-Tu, we performed LFIA with *P. aeruginosa* PAR1-spiked milk (OD = 1.0). Moreover, to prove that our LFIA did not detect *E. faecalis*, we used *an E. faecalis* ATCC 29,212-spiked milk (OD = 1.0) assay. In both, no red line appeared on the test.

**Table 1:** Bacterial density used at all stages of the study. Legend: OD600 – optical density, measured at a wavelength of 600 nm; CFU – colony forming unit

OD_600_	CFU/ml	CFU/well
OD_600_ = 2.0	~ 6.0 × 10^9^	~ 6.0 × 10^8^
OD_600_ = 1.0	~ 3.0 × 10^9^	~ 3.0 × 10^8^
OD_600_ = 0.5	~ 1.5 × 10^9^	~ 1.5 × 10^8^
OD_600_ = 0.1	~ 3.0 × 10^8^	~ 3.0 × 10^7^
OD_600_ = 0.01	~ 3.0 × 10^7^	~ 3.0 × 10^6^
OD_600_ = 0.001	~ 3.0 × 10^6^	~ 3.0 × 10^5^

**Table 2:** LFIA parameters determined for two approaches: (1) a monoplex diagnostic assay for the detection of *S. uberis* in bovine milk samples and (2) a multiplex assay for the detection of *S. uberis*, *S. agalactiae*, and *S. aureus* in bovine milk samples

LFIA assay	SU detection	SU/GBS/SA detection
Sensitivity (95% CI)	43.59% (27.81 − 60.38%)	43.28% (31.22 − 55.96%)
Specificity (95% CI)	89.02% (83.21 − 93.36%)	95.59% (90.64 − 98.36%)
Positive predictive values (PPV) (95% CI)	0.49 (0.31–0.66)	0.83 (0.66–0.93)
Negative predictive values (NPV) (95% CI)	0.87 (0.81–0.92)	0.77 (0.7–0.83)
Positive likelihood ratio (PLR) (95% CI)	3.97 (2.26–6.98)	9.81 (4.28–22.48)
Negative likelihood ratio (NLR) (95% CI)	0.63 (0.48–0.84)	0.59 (0.48–0.73)
Accuracy (ACC) (95% CI)	80.3% (74.15 − 85.53%)	78.33% (72.02 − 83.79%)
Diagnostic odds ratio (DOR) (95% CI)	6.27 (2.82–13.95)	16.54 (6.39–42.77)

## Discussion

Bovine mastitis constitutes a serious issue worldwide, creating substantial losses for cattle breeders. These losses stem from lower milk quality, lower milk yields, and the need to isolate sick animals, during which time they do not produce milk and require appropriate treatment [38–40]. Therefore, a rapid, precise, accessible, affordable assay that can identify the bacterial species causing mastitis would help a veterinarian quickly make a diagnostic decision and introduce a targeted antibiotic therapy during a single visit to the barn. Therefore, lateral flow immunoassay would fulfill the gap in the veterinary diagnostic market. To our best knowledge, there is currently no test that meets these requirements.

Our study showed that the assay based on mAb-anti-EF-Tu for the detection *of S. uberis* in a bovine milk sample, the most common species responsible for this disease, demonstrated a sensitivity of 43.59%; however, the specificity was almost 90% and the accuracy was over 80%, which is also satisfactory.

Studies on LFIA for a rapid mastitis diagnosis, broadly described in the literature, are mostly focused on the assay dedicated to detecting *S. aureus* and *E. coli*. Nagasawa et al. based their assay for detecting *S. aureus* in milk samples on ribosomal protein (RP)-L7/L12; the results were very promising [41]. Sayed et al. studied a lateral flow device for rapid simultaneous multiple detections of *E. coli*, *S. aureus*, *Klebsiella pneumoniae, Streptococcus agalactiae*, and *Streptococcus pyogenes*; the sensitivity, specificity, and accuracy determined in comparison with the gold standard were 95.29%, 80.00%, and 95.51% for *E. coli*; 97.50%, 78.57%, and 94.68% for *S. aureus*; 93.61%, 83.33%, and 92.45% for *K. pneumoniae*; 89.19%, 80.00%, and 87.23% for *S. agalactiae*; and 83.33%, 80.00%, and 82.76% for *S. pyogenes*, respectively [42]. Fang et al. developed LFIA for the detection of *Salmonella* spp. and achieved very high sensitivity and selectivity values, reaching 100% and 99%, respectively [43]. In turn, Bautista et al., whose assay was dedicated to the identification of Salmonella sp. in poultry, reported a significantly lower sensitivity value (12.3%) in comparison with other assays, though the specificity was 100% [44]. The variation in the assay parameters is notable and may result from a number of factors. Nevertheless, the most important indicator seems to be the specificity and quality of the antibodies that recognize particular antigens.

Another parameter that may impact the positive results is the number of bacteria in the samples. In our study, the mean bacterial concentration was ≥ 10^2^ CFU/mL. In turn, Nagasawa et al. obtained such high sensitivity (100%) and specificity (91.9%) for a CFU/mL of ≥ 10^4^ [41]. Sayed et al. indicated that positive results were possible with a minimum of 10^3^ CFU/mL [42]. Kumar et al. also determined the cut-off point for CFU at 10^3^ CFU/mL, even though that study was dedicated to the development of an ELISA assay for *Salmonella typhi* in food and water samples [45]. In turn, Wiriyachaiporn et al.’s assay for *S. aureus* detection [46] and Jung et al.’s LFIA for detection of *E. coli* O157: H7 in bovine feces [47] set cut-off points at 10^6^ CFU/mL and 10^5^ CFU/mL, respectively. This may explain the lower sensitivity in our studies, as the maximum CFU for *S. uberis* among our milk samples was 10^2^ CFU/mL. It is worth noting that none of these tests were dedicated to detecting *S. uberis*; therefore, particular CFU values can vary between species.

Another explanation for the lower sensitivity values in comparison with the above-mentioned papers is a lack of pre-incubation. Because the purpose of our assay is to be performed quickly, on-site, and without other bacteriological methods, introducing prepared materials would limit the assay’s ability to be performed by an untrained stakeholder, such as a farmer. As a result, the wait time for the results is 15 min. The introduction of a pre-incubation step may extend the wait time for a diagnosis to 4 or even 24 h [45, 48]. Nevertheless, this step should be considered in future studies, as it may improve the results even tenfold [45], even though it limits its usage for laboratory conditions.

EF-Tu is a conservative moonlight protein which is present in most bacteria [21–23]. However, our previous study on the detection of this protein using serum samples from cows with mastitis of various etiologies did not indicate cross-reactivity among other Gram-positive cocci [17]. For this reason, and the knowledge of its immunoreactivity, we decided to choose this target for further investigation. The results obtained through ELISA and Western blotting consistently indicated the occurrence of cross-reactions among *S. uberis*, *S. agalactiae*, and *S. aureus*. The analysis of raw data obtained through LFIA allowed us to consider another approach of using the assay to detect one or three species. The parameters determined for the multiplex variant of LFIA for detecting *S. uberis*, *S. agalactiae*, and/or *S. aureus* were also very promising (specificity of 95.59%, sensitivity of 43.28%, and accuracy of 78.33%), and even though unambiguous detection of bacterial species is impossible, it could direct veterinarians to the appropriate treatment or set a path for further diagnostics.

Our study demonstrated the cross-reactivity of the monoclonal antibody anti-EF-Tu with certain Gram-positive cocci. This characteristic of our LFIA test is precious from a veterinary perspective. It enables the identification of etiological agents such as *S. uberis, S. agalactiae*, or *S. aureus* while excluding *Enterococcus* spp. and Gram-negative rods. This is significant because the most frequently isolated pathogens in mastitis cases are Gram-positive and catalase-negative cocci, including *S. agalactiae* and *S. uberis*, which account for 47% of cases. *S. aureus* species constitute the second-largest group of pathogens, at 37%. *Enterococcus* spp. were found in 4% of bacterial mastitis cases, and Gram-negative rods in about 9.4% of cases [49].

The primary antibiotics for treating bacterial mastitis are penicillins (benzylpenicillin, oxacillin, and cloxacillin), which are effective against *S. uberis, S. agalactiae*, and *S. aureus*. However, *Enterococcus* spp. often show resistance to penicillins, except for *E. faecalis*, which is sensitive to ampicillin or amoxicillin. Conversely, Gram-negative rods frequently demonstrate resistance mechanisms to beta-lactam antibiotics. In these cases, treatment options include fluoroquinolones like enrofloxacin or marbofloxacin and third- and fourth-generation cephalosporins. In summary, this illustrates the practical utility of the cross-reactivity feature of our LFIA test in identifying and guiding treatment options for bacterial mastitis.

We hypothesize that a different approach of mAb obtaining [42] or using a mixture of mAb directed against several conservative and species-specific antigens would improve this parameter. Another solution to improve the results could include an additional step of optimization of the buffer composition in used antibodies. Moreover, choosing more specific protein antigens from bacteria causing mastitis can also be considered. Another idea is based on using more specific types of antigens, e.g., bacterial extracellular polysaccharides rather than protein antigens, which could result in a more specific binding for monoclonal antibodies. We hope that it will encourage further studies to develop this assay.

## Conclusions

To our best knowledge, no such assay LFIA has been developed for *S. uberis* detection in mastitic milk samples. The specificity and accuracy values, as well as their confidence intervals, can be considered high; however, we are aware that the sensitivity should be improved. Even though the results are pioneering, further studies are undoubtedly required.

## Materials and methods

### Characterization of EF-Tu protein as a bacterial antigen

The method of detection and characterization of EF-Tu specific to *S. uberis* was described in our previous study [17]. In brief, isolated bacterial surface proteins (*n* = 11) were separated in SDS-PAGE and immunoreactive proteins were detected in the presence of serum samples obtained from SU-positive animals (*n* = 6) or from healthy animals (control group; *n* = 12). Blood samples for CM serum isolation were collected into a clot activator tube (S-MONOVETTE) through a single jugular venipuncture during routine diagnostic tests to identify mastitis. Blood samples of the animals in the control group were collected by veterinarians during routine examinations to determine typical biochemical parameters in healthy animals. Next, the samples were kept for 3 h at room temperature until a clot was formed. Then, the serum was collected into sterile tubes (BD) using a pipette after centrifuged (2000 x g) for 15 min at 4 ˚C. The clear supernatant was divided into sterile Eppendorf tubes and stored at -80 °C until further analyses. Subsequently, bands of interest (four for *S. uberis*), defined as common among individual species and not present (or barely reactive) among other species, were cut out from the multiple gels and subjected to further analysis, including protein identification by the MALDI-ToF method using an Ultraflextreme MALDI TOF/TOF spectrometer (Bruker, Bremen, Germany) and flexControl 3.3 software (Bruker, Bremen, Germany). Next, the proteins identified by sequencing were subjected to bioinformatic analysis, which included investigating their structure, function, and immunogenicity by predicting subcellular localization, signal peptides, antigenicity, and classical and non-classical proteins. In addition, selected proteins were subjected to linear B-cell epitope identification, a protein data bank (PDB) model search, and identification of conformational B-cell epitopes. Thus, the analysis, which combined *in silico* and in vitro studies with a literature review and analysis of the results from our previous study on EF-Tu [17, 37], allowed us to consider this protein as an appropriate antigen candidate in LFIA for bovine mastitis diagnosis.

### Isolation of native proteins

To examine the immunoreactivity of native proteins and of whole live bacteria with mAb-anti-EF-Tu, previously characterized *S. uberis* strains (*n* = 2) [17], were suspended in phosphate buffered saline (PBS; ABO) and then underwent protein isolation. According to the first protocol [50], a bacterial suspension with an optical density OD_600_ = 1.0 in a volume of 1 mL was centrifuged for 10 min at 4500 RPM, and subsequently suspended in 200 µL of lysis buffer consisting of 0.2% SDS (Thermo Fisher), 0.1% Triton X-100 (PolAura), 50 µg/mL lysozyme (Merck), and 1 µL of benzonase (Merck). The lysates were incubated at 25 ˚C for 30 min and subsequently at 42 ˚C for 1 h with constant, vigorous shaking (700 RPM).

In the second procedure carried out according to Banner et al. [51] with slight modifications, 2 mL of bacterial suspension with an optical density OD_600_ = 2.0 was centrifuged for 10 min at 4500 RPM, and subsequently suspended in 150 µL of lysis buffer A, consisting of 100 µg/mL lysozyme, 1 µL of benzonase and 50 U of mutanolysin (A&A Biotechnology). After 30 min of preincubation at 25 ˚C with constant, vigorous shaking (700 RPM), 150 µL of lysis buffer B, consisting of 0.4% SDS, was added to each sample and incubated at 42 ˚C for 30 min with constant, vigorous shaking (700 RPM). After analyzing the quantity of protein bands obtained in the isolation according to these procedures, we chose the second procedure using mutanolysin.

The quantity of isolated proteins was determined using the Bradford assay. Therefore, 10 µL of bacterial lysate was incubated with 250 µL of the Bradford reagent (Thermo Scientific Chemicals), and the samples were incubated in darkness for 10 min. Absorbance values for the samples were measured at a wavelength of 595 nm using SpectraMax® i3 multi-mode microplate reader and the results were referred to the standard curve obtained by BSA dilutions in a range of 0.00–1.50 mg/mL.

### ELISA

Enzyme linked immunosorbent assay (ELISA) was carried out in order to determine the monoclonal antibodies (Ab) titer as a component of the LFIA as well as antigens detected in the test specimen. In the first stage, the cross-reactivity between the components of sterile milk in the presence of monoclonal antibodies (mAb-anti-EF-Tu, Biolim S. A.) and the reactivity between recombinant EF-Tu (Biolim S. A.) and monoclonal antibodies (anti-EF-Tu) were evaluated.

To this end, wells of a 96-well MaxiSorp plate (Merck) were coated with 100 µL of recombinant EF-Tu protein at a concentration of 2.5 µg/mL or 100 µL of sterile milk. The plates were then incubated at 4 ˚C for 16 h. Subsequently, unbound antigens were washed three times in Tris buffered saline supplemented with 0.01% Tween 20 (TBST, Merck). The free surface on the wells was blocked with 5% skim milk (Merck) in PBS for 2 h, then washed three times with TBST buffer. The mAb-anti-EF-Tu antibodies were diluted in 2% skim milk (Merck) in PBS in decreasing concentrations between 1:50 and 1:51,200,000 and 100 µL of an appropriate Ab solution was added to each well and incubated at 4 ˚C for 2 h. Unbound Abs were washed three times in TBST buffer. Subsequently, 100 µL of horseradish peroxidase-conjugated goat anti-mouse antibodies (Thermo Fisher) diluted (1:5000 ratio) in 2% skim milk in PBS was added to the wells and incubated at 4 ˚C for 1 h. The wells were washed three times with TBST buffer. The product of the enzymatic reaction was visualized by incubation with TMB solution (Merck) at room temperature for 20 min. The reaction was stopped by adding 50 µL of 1 N H_2_SO_4_ (Merck). Absorbance was measured at wavelengths of 450 and 570 nm by a SpectraMax® i3 multi-mode microplate reader (Syngen, Taipei City, Taiwan). The results of the three measurements for each sample were processed in GraphPad Prism 9 by Dotmatics and are expressed as mean absorbance values. The Ab titer was defined as the first decrease in the absorbance after the plateau.

### Cellular ELISA

Cellular ELISA (cELISA) was carried out to investigate the binding capacity of the antibodies to whole bacterial cells and to determine an appropriate antigen concentration for Western blotting. The procedure was carried our according to Kawka et. al’s procedure [52].

*S. uberis* strains were cultivated on chocolate blood solid medium (BD Difco™ GC agar + BD BBL™ Hemoglobin, Bovine, Freeze-Dried) and incubated at 37 °C for 48 h in a 5% CO_2_ atmosphere. Next, the bacteria were suspended in PBS and adjusted to an optical density of OD600 = 1.0. Then, 100 µL of 4% formaldehyde was added to 900 µL of the bacterial suspension. Next, 100 µL/well of fixed bacteria were placed on a 96-well plate and incubated at 37 °C overnight until the wells were dried. The remaining steps of the procedure were the same as for the ELISA procedure described above.

### SDS-PAGE and Western blot

To evaluate the binding capacity of the mAb to the bacterial cell, the bacterial lysates were separated by SDS-PAGE. Bacterial lysates diluted in PBS to a final protein concentration of 20 µg/mL were mixed with a sample buffer (Thermo Fisher), incubated at 95 °C for 5 min, and separated by SDS-PAGE in four replicates.

As a positive control, rEf-TU protein obtained from *S. uberis* for antibody (mAb-anti-EF-Tu) binding capacity was used. The lysates from Gram-positive bacteria other than *S. uberis* in performed Western blotting were used since the elongation factor Tu (EF-Tu) is among the most abundant proteins in bacterial cells. In parallel, to exclude nonspecific binding of antibodies, *P. aeruginosa*, *K. pneumoniae* and *E. coli* were included. In turn, the purpose of using whole milk samples with verified microbiological status was to exclude mAb cross-react with milk samples.

After separation, the proteins were transferred onto nitrocellulose membranes for Western blotting. Each experiment was performed in three independent replicates. Following 1.5 h of transfer at 100 V in cooled conditions, the membranes were blocked with Blocking Solution (Thermo Fisher) for 1 h with shaking; the membranes were subsequently coated (separately) with anti-EF-Tu Abs in the 1:50,000 titer in 2% skim milk PBS solution and incubated at 4 °C for 16 h with constant shaking. After incubation, the membranes were washed three times for 10 min using TBST. Next, the membranes were coated with HRP-conjugated goat anti-mouse Abs (Invitrogen) in a titer of 1:5,000 and incubated at 4 °C for 2 h with constant shaking. The membranes were washed three times for 10 min using TBST buffer. Subsequently, TMB (Thermo Fisher) was added onto the membranes until completely coated and they were incubated at 4 °C in darkness for 20 min. The prepared membranes were then visualized by ProXima 2750 (T) (Isogen Life Science).

### Preparation of monoclonal antibodies conjugated with gold nanoparticles

### Production of gold nanoparticles

Gold nanoparticles (AuNps) with a diameter of 30 nm were synthesized in a chemical reaction involving a reduction of tetrachloroauric (III) acid (Thermo Fisher) using sodium citrate (Chempur). Before starting the reaction, the laboratory glassware was cleaned chemically using aqua regia solution of 3 parts hydrochloric acid (Chempur) to 1 part nitric (V) acid (Chempur). Production of gold nanoparticles was carried out according to Zhang et al’s procedure [53].

To synthesize the nanoparticles, the 0.01% tetrachloroauric (III) acid solution (Thermo Fisher) was brought to a boil. Then, 1 mL of 1% sodium citrate (Merck) was added and boiled for another 10 min with constant, vigorous stirring. The color of the solution changed from yellow through gray to an intense burgundy color during the reaction. To prevent excessive evaporation, the reactions were completed using Graham condenser. After the reaction finished, the solution was cooled down to room temperature. Next, 100 mL of gold nanoparticle solution was prepared and subjected to UV-VIS and dynamic light scattering analysis in five replicates to determine their size and to estimate the properties of the colloid system (monodispersity). As a result, 100 mL of stable monodisperse gold nanoparticle solution with a diameter of 30 nm was obtained.

### Conjugation of monoclonal antibodies and gold nanoparticles

The resulting AuNps and mAb-anti-EF-Tu (Biolim) were conjugated according to Seele et al’s procedure [54]. In the first step, the pH of aqueous AuNps solution was set to 7.5 using 0.2 M K_2_CO_3_ (potassium carbonate, Merck). Subsequently, a microscale stability test of mAb conjugation with AuNps was performed. A series of mAb dilutions – 40, 20, 10, 5, 2.5, and 1.25 µg/mL – were prepared using ultrapure water buffered with K_2_CO_3_ (Merck) at a pH of 7.5. The above concentrations of mAb were added to 100 µL of AuNps and the complexes were incubated at room temperature for 1 h to bind the mAbs with AuNps. Following the incubation, 50 µL of 1 M NaCl was added and incubated at room temperature for 30 min. UV-VIS measurements in a wavelength range of 450–600 nm were performed with a SpectraMax® i3 multi-mode microplate reader.

In the next step, mAb and AuNps were conjugated on a larger scale, based on the above optimization. Therefore, dilutions of mAbs-anti-EFTu were prepared in concentrations of 10 µg/mL in 2 mL of borate buffer (Merck) at a pH of 8.5. The resulting dilutions were added to 10 mL of AuNps and incubated at 4 °C for 1 h. After incubation, 1.5 mL of 10% BSA (Merck) solution, prepared from a 20 mM borate buffer, was added and incubated at 4 °C for 15 min. Subsequently, the complexes were centrifuged at 2,500 × g at room temperature for 30 min and washed twice with 20 mM borate buffer containing 1% BSA. The pellet was finally resuspended in 1 mL of 20 mM borate buffer and added to 1 mL of suspension buffer containing 3% sucrose (Merck), 0.6 M NaCl, 0.2% Tween-20 (PolAura), and 2% BSA, prepared in 20 mM borate buffer (pH = 8.0).

### Lateral flow immunoassay

The following materials were used in the construction of the lateral flow immunoassay: (1) a sample pad, composed of fiberglass material (Glass Fiber Diagnostic Pad, Merck), which was soaked in a buffer containing PBS, with 0.2% Tween-20 and 0.1 M NaCl, then dried at 50 °C for 30 min; (2) a conjugate pad, composed of fiberglass material (Glass Fiber Diagnostic Pad, Merck), on which AuNP conjugated with mAbs (OD = 4.0) resuspended in a 20 mM borate buffer with a pH of 8.0, 3% sucrose, 0.6 M NaCl, 0.2% Tween-20, and 2% BSA was applied in the amount of 20 µL/cm; (3) a nitrocellulose membrane composed of nitrocellulose (Hi Flow Plus HF 90 membrane card, Merck); and (4) an absorbent cellulose pad (Cellulose Fiber Sample Pad, Merck) [53, 55].

**Fig. 9:**
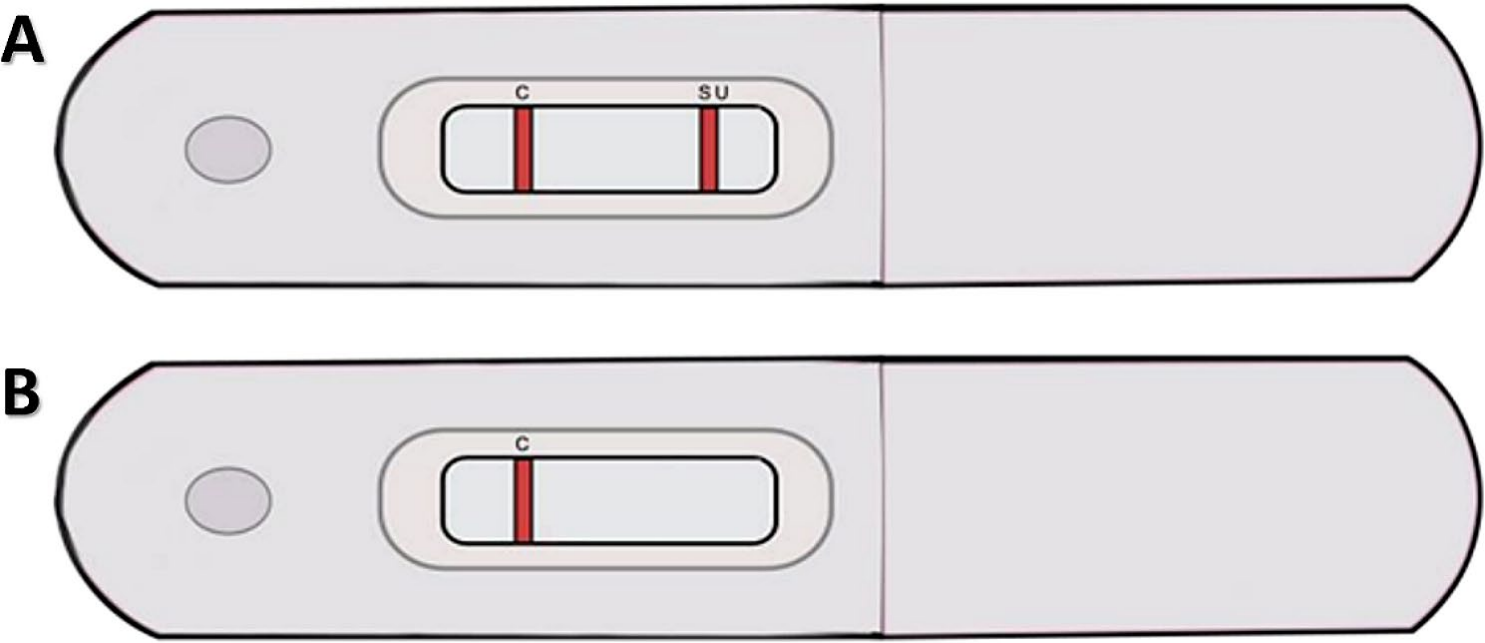
Schematic of the LFIA assay for the detection of *S. uberis* (SU) in milk samples. **A** – example of a positive test result; **B** – example of a negative test result. Legend: **C** – control line, indicating the correct performance of the test; SU – *S. uberis*, test line, indicating the correct performance of the test and the presence of *S. uberis* in the tested milk sample

There were two test lines applied to the nitrocellulose membrane: *(A) the test line* was mAb against bacterial proteins at a concentration of 3 mg/mL and *(B) the control line* was mAb against mouse antibodies (Thermo Fisher) at a concentration of 1 mg/mL (Fig. 9).

Both lines were applied by an automated lateral flow reagent dispenser (ClaremontBio) at 1 µL/cm, with a 0.75-cm gap between them. Next, the membranes were blocked with 1% BSA until the whole membrane surface was fully coated and subsequently dried at 37 °C for 2 h. The lateral flow immunoassay was assembled as follows. The sample pad overlapped with its surface onto the conjugate pad, which overlapped with the start of the nitrocellulose membrane. The absorbent pad was placed at the end of the nitrocellulose membrane to absorb any excess liquid. Test bands with a width of 30 mm were then cut out from the membranes thus prepared. In the first stage, the lateral flow tests were performed using 100 µL of EF-Tu recombinant protein, diluted to a concentration of 0.5 mg/mL, and PBS as a negative control.

Next, tests using the reference *S. uberis* strain were performed. The bacteria suspensions were prepared in 0.85% NaCl to OD = 1.0 (3 × 10^8^ CFU/test), OD = 0.1 (3 × 10^7^ CFU/test), OD = 0.1 (3 × 10^6^ CFU/test), and OD = 0.001 (3 × 10^5^ CFU/test). A negative control (0.85% NaCl) was included and LFIA was developed by 100 µL of suspension or saline.

Obtained results were then interpreted by three independent people analyzed the tests. Moreover, the LFIA results can also be read using a lateral flow assay reader.

### Collection and characterization of milk samples

Milk samples (*n* = 203) were collected from udder quarters of 203 Holstein Friesian cattle in age ranging from 2 to 6 years old in the routine course of milking and were divided into two groups. The study group consisted of 100 milk samples from cattle with clinical signs of mastitis (CM) and the following symptoms: swelling, heat, hardness, redness, or pain of the udder; watery appearance, flakes, clots, or pus in the milk; and increased body temperature or lack of appetite. The control group consisted of 103 milk samples from cattle with no clinical symptoms of mastitis. The samples were collected from different centers in Poland: Omniwet Veterinary Clinic in Orzesze (southwestern Poland) (*n* = 47), Egida Veterinary Clinic in Wizna (northeastern Poland) (*n* = 117), and the University Centre for Veterinary Medicine Institute of Veterinary Sciences in Krakow (southern Poland) (*n* = 39). The cows involved in the investigation came from different cowsheds, both free-range and those confined to cowsheds. According to the 1st Local Ethics Committee for Animal Experimentation at Jagiellonian University Medical College in Krakow, Poland, which oversees animal experimentation, determined that formal consent for this research project was unnecessary. This decision was based on the fact that the samples were collected as part of standard veterinary examinations.

The herds were assessed by the Polish Federation of Cattle Breeders and Milk Producers, which requires monthly reports of milk parameters, such as the number of somatic cells per cow milked. The health status of the animals in the control group was determined based on physical parameters, such as the absence of oedema or redness of the udder, as well as biochemical parameters by determining the number of somatic cells by California Mastitis Test (CMT). Animals with somatic cells > 100.000/ml and with clinical signs were classified as sick, whereas the lack of symptoms was a basis for classifying the animals as healthy. Moreover, sample classification was carried out according to the guidelines of National Mastitis Council (NMC), following which, five or more colonies of an environmental species were considered as an infection while three or more species in one sample was treated as a contamination.

To validate the lateral flow immunoassay, first milk samples were characterized in order to identify the etiological factors that caused mastitis in particular udder quarters and find out the microbiological status of control milks. For this purpose, 100 µl of each milk sample was inoculated on CHROMagar™ Mastitis (included: CHROMagar™ Mastitis Gram-Positive (MS252/P) and CHROMagar™ Mastitis Gram-Negative (MS252/N) medium, and then cultured at 37 ˚C under aerobic conditions for 18 h.

The milk samples were delivered to the Department of Microbiology at the Jagiellonian University Medical College in Krakow and to the Department of Immunology and Infectious Biology, Faculty of Biology and Environmental Protection at the University of Łódź on dry ice under deep-freeze conditions. They were stored at -80 ˚C until further analyses.

The following species, which are the most common etiological agents in bovine mastitis, were included as a cross-reactivity control: *E. coli* EC1, *K. pneumoniae* KP1, *P. aeruginosa* PAR1, *S. aureus* SA1, *S.s aureus* ATCC 29,213, *E. faecalis* ATCC 29,212, *S. agalactiae* GBS1, *S. uberis* SU1, and *S. uberis* SU2. For all species, there were 10^8^ CFU/mL. Four bacterial concentrations were studied in the investigation. The optical density values used in this study are shown in Table 1.

### LFIA validation

The validation of the newly constructed LFIA prototype was a rigorous process. Blinding data on the status of the analyzed milk was used to determine the status of the collected milk. Using the parameters determined in the previous steps, the mAb-anti-EF-Tu LFIA test for detection of *S. uberis* was validated with 203 milk samples; the results were compared with a reference method (microbiological evaluation). These results encouraged us to consider the LFIA assay for two diagnostic approaches: monoplex LFIA for *S. uberis* in mastitis milk diagnosis and multiplex LFIA for *S. uberis*, *S. agalactiae*, and/or *S. aureus* in mastitis milk diagnosis. Therefore, these two approaches were subjected to statistical analysis. The LFIA was classified as positive when both: control and test lines were detectable visually as red bands.

### Statistical analysis

To assess the diagnostic reliability of the newly developed test, its sensitivity, specificity, positive predictive value (PPV), negative predictive value (NPV), positive likelihood ratio (PLR), negative likelihood ratio (NLR), diagnostic odds ratio (DOR), and accuracy (ACC) were calculated along with 95% confidence intervals (CI). These key diagnostic parameters commonly used in literature to assess the performance of diagnostic tests were used for calculation for both diagnostic approaches: a monoplex diagnostic test for detecting *S. uberis* in bovine milk samples and a multiplex test for detecting *S. uberis, S. agalactiae*, and *S. aureus* in bovine milk samples. Culturing on CHROMagar™ Mastitis medium was taken as our gold standard. The test parameters were evaluated using PQStat 1.8.6.102 software.

### Electronic supplementary material

Below is the link to the electronic supplementary material.

Supplementary Material 1

Supplementary Material 2

Supplementary Material 3

Supplementary Material 4

## Data Availability

No datasets were generated or analysed during the current study.

## Abbreviations

ACC: Accuracy
ATCC: American Type Culture Collection
AuNPs: gold nanoparticles
BSA: bovine serum albumin
cELISA: cellular enzyme linked immunosorbent assay
CFU: colony forming unit
CI: confidence interval
CM: clinical mastitis
DOR: diagnostic odds ratio
EF: Tu–elongation factor thermo unstable
ELISA: enzyme linked immunosorbent assay
GBS: group B streptococcus
HRP: horseradish peroxidase
LFIA: lateral flow immunoassay
mAb: EFTu–monoclonal anti–EFTu antibodies
MALDI: ToF–matrix–assisted laser desorption/ionization time of flight
NAS: non–aureus Staphylococci
NLR: negative likelihood ratio
NPV: negative predictive values
OD: optical density
PLR: positive likelihood ratio
PPV: positive predictive values
SCC: somatic cell count
SDS: sodium dodecyl sulfate
SDS: PAGE–sodium dodecyl sulfate–polyacrylamide gel electrophoresis
PDB: protein data bank
RPM: round per minute
TBST: Tris buffered saline with Tween 20
TMB − 3,3′,5,5′: Tetramethylbenzidine
