# Supplementary figures and images for: Development of a prototypic, field-usable diagnostic tool for the detection of gram-positive cocci-induced mastitis in cattle
Source: BMC Vet Res. 2024 May 2;20:169. doi: 10.1186/s12917-024-04028-5 (PMC11064325; doi:10.1186/s12917-024-04028-5)

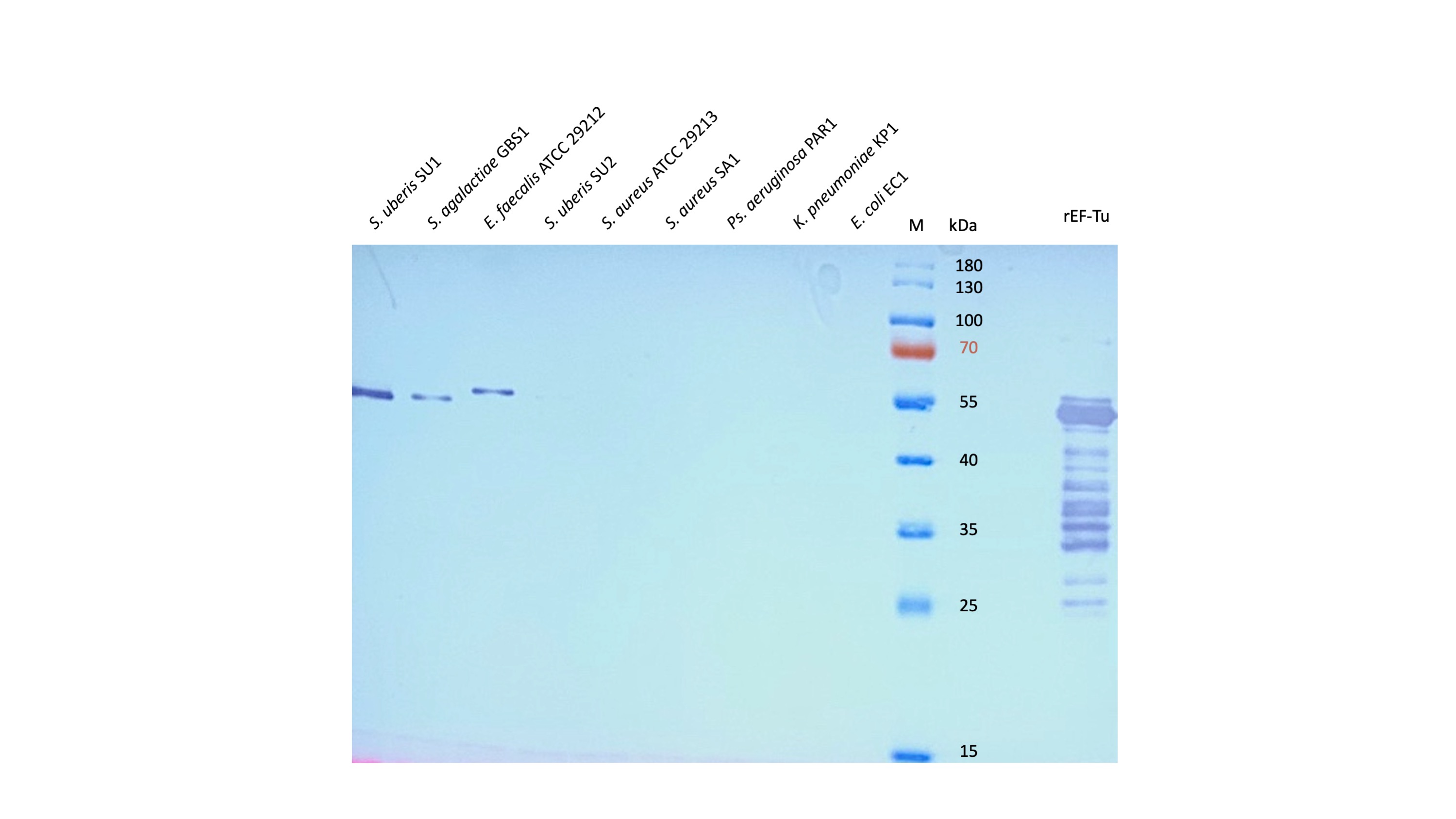

Supplement: Supplementary file 1 — Supplementary Material 1 [file 12917_2024_4028_MOESM1_ESM.jpg]

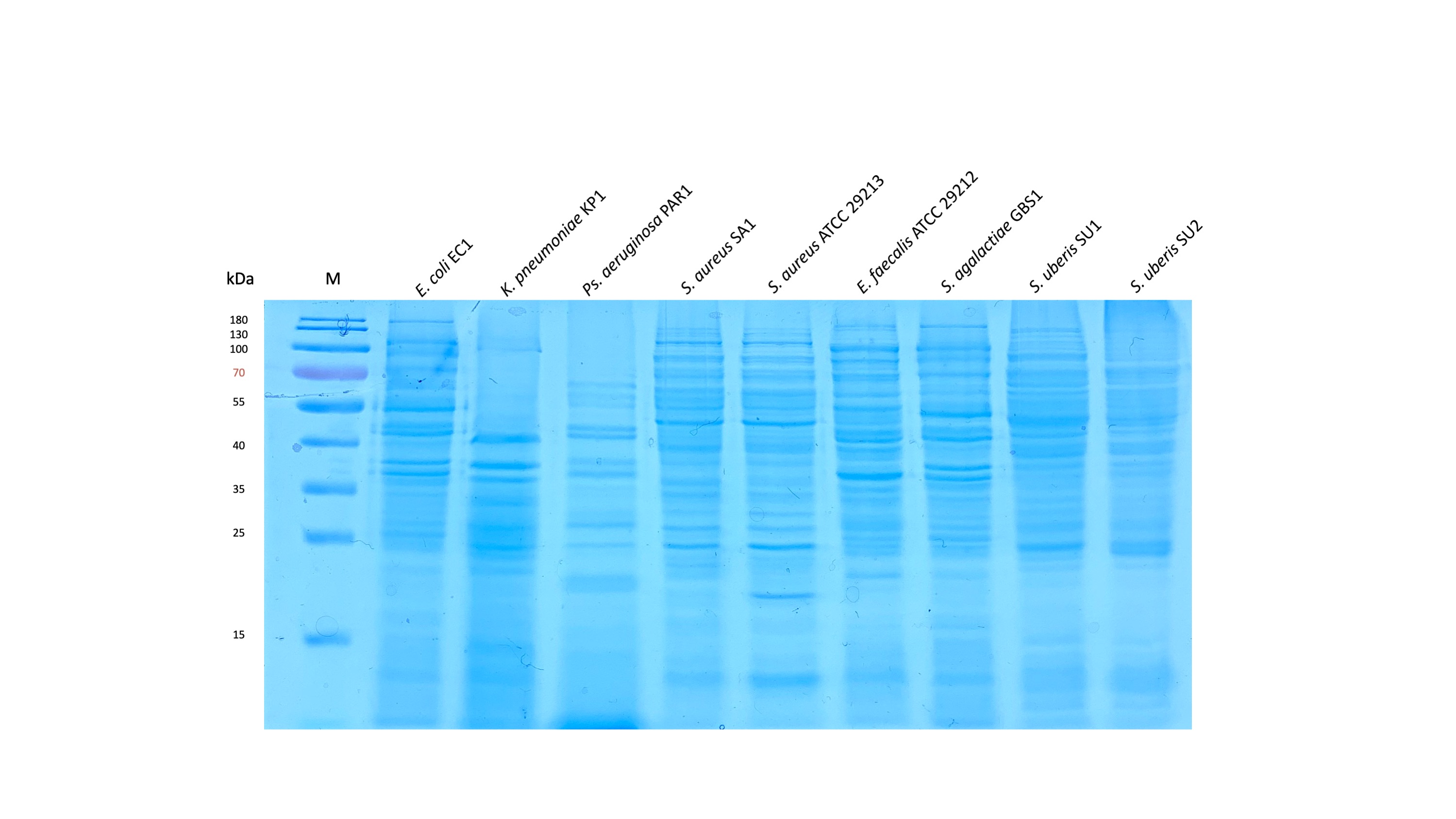

Supplement: Supplementary file 2 — Supplementary Material 2 [file 12917_2024_4028_MOESM2_ESM.jpg]

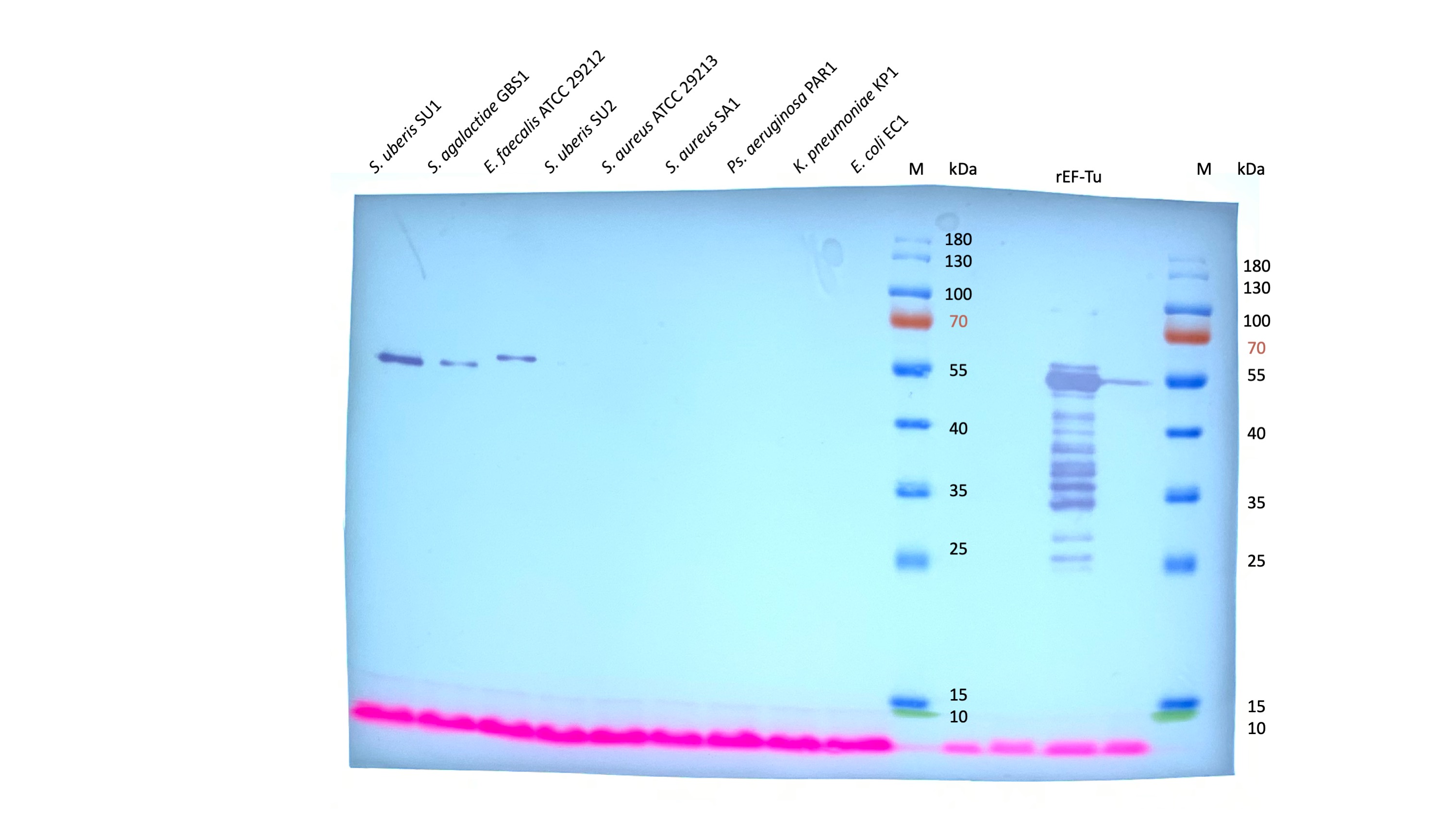

Supplement: Supplementary file 3 — Supplementary Material 3 [file 12917_2024_4028_MOESM3_ESM.jpg]

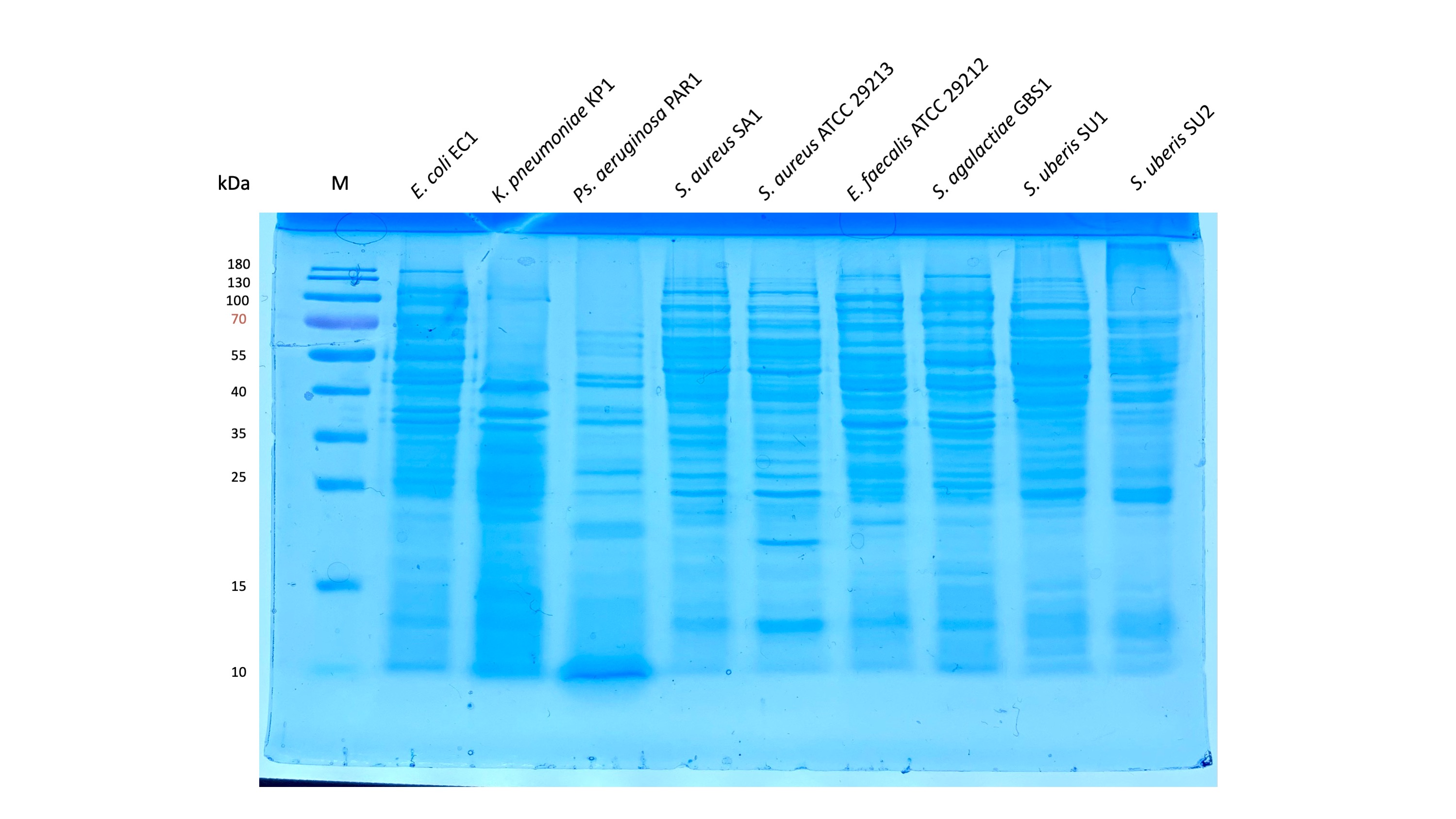

Supplement: Supplementary file 4 — Supplementary Material 4 [file 12917_2024_4028_MOESM4_ESM.jpg]
